# Heterozygosity mapping for human dominant trait variants

**DOI:** 10.1002/humu.23765

**Published:** 2019-04-24

**Authors:** Atsuko Imai‐Okazaki, Yi Li, Sukanya Horpaopan, Yasser Riazalhosseini, Masoud Garshasbi, Yael P. Mosse, Di Zhang, Isabelle Schrauwen, Aarushi Sharma, Cathy S. J. Fann, Suzanne M. Leal, Mark Lathrop, Jurg Ott

**Affiliations:** ^1^ Diagnostics and Therapeutics of Intractable Diseases Intractable Disease Research Center Graduate School of Medicine Juntendo University Tokyo Japan; ^2^ Division of Genomic Medicine Research Medical Genomics Center National Center for Global Health and Medicine Tokyo Japan; ^3^ Laboratory of Statistical Genetics Rockefeller University New York New York; ^4^ School of Statistics Shanxi University of Finance and Economics Taiyuan China; ^5^ Department of Anatomy Faculty of Medical Science Naresuan University Phitsanulok Thailand; ^6^ McGill University and Genome Québec Innovation Centre Montréal Québec Canada; ^7^ Department of Medical Genetics Faculty of Medical Sciences Tarbiat Modares University Tehran Iran; ^8^ Division of Oncology and Center for Childhood Cancer Research Children's Hospital of Philadelphia Department of Pediatrics Perelman School of Medicine at the University of Pennsylvania Pennsylvania Philadelphia; ^9^ Center for Statistical Genetics Baylor College of Medicine Houston Texas; ^10^ Institute of Biotechnology Amity University Gwalior Madhya Pradesh India; ^11^ Institute of Biomedical Sciences Academia Sinica Taipei Taiwan

**Keywords:** ALSPAC, computer simulation, gene mapping, genetic association analysis, sequence variants

## Abstract

Homozygosity mapping is a well‐known technique to identify runs of homozygous variants that are likely to harbor genes responsible for autosomal recessive disease, but a comparable method for autosomal dominant traits has been lacking. We developed an approach to map dominant disease genes based on heterozygosity frequencies of sequence variants in the immediate vicinity of a dominant trait. We demonstrate through theoretical analysis that DNA variants surrounding an inherited dominant disease variant tend to have increased heterozygosity compared with variants elsewhere in the genome. We confirm existence of this phenomenon in sequence data with known dominant pathogenic variants obtained on family members and in unrelated population controls. A computer‐based approach to estimating empirical significance levels associated with our test statistics shows genome‐wide *p*‐values smaller than 0.05 for many but not all of the individuals carrying a pathogenic variant.

## 1 INTRODUCTION

Thirty years ago, the concept of homozygosity mapping (HM) was published (Lander & Botstein, 1987) and has since been highly influential. For rare autosomal recessive traits due to a homozygous genetic variant, when the two susceptibility alleles at the disease variant are inherited as two copies of a single disease allele from a common ancestor, variants in the DNA region surrounding the disease variant also tend to be homozygous. Thus, researchers may simply look for extended runs of homozygosity (ROH) in affected individuals, or compare lengths of ROHs between affected and unaffected individuals, to identify genomic regions likely to harbor a recessive disease gene (Lander & Botstein, 1987). HM has been hugely successful for the mapping and identification of recessive disease variants, particularly with massively parallel sequence data (Morrow et al., 2008; Pippucci, Magi, Gialluisi, & Romeo, 2014; Stingl et al., 2017).

Some 10 years later, a method called linkage disequilibrium (LD) mapping was developed for the mapping of heterogeneous recessive traits (Feder et al., 1996). This method was critically reviewed and generalized to other disease models (Nielsen, Ehm, & Weir, 1999), but it does not seem to have been used much since.

Klein et al. (1998) extended the LD mapping approach to dominant traits and demonstrated that markers in the vicinity of a (rare) dominant trait variant should show a heterozygote excess. For a given marker, these authors developed a *χ* ^2^ test statistic. However, in their data on focal dystonia, *χ* ^2^ turned out to be zero even though other data (Leube et al., 1997a, 1997b; Leube et al., 1996) had shown evidence for disequilibrium for the same markers and trait. The proposed approach (Klein et al., 1998) does not seem to have been used further.

Here we describe a novel method, heterozygosity analysis (HA), applicable to inherited dominant disease variants. It is based on the rate of heterozygosity (vs. homozygosity), which tends to be elevated in sequence variants surrounding an inherited dominant disease variant. Such a variant is passed on a chromosome from a parent to offspring within a (possibly short) segment of DNA in which variants have not experienced recombination with the disease variant. The other chromosome, however, is likely to have undergone recombination throughout its length. We show that this discrepancy leads to a higher rate of heterozygosity at variants in the immediate vicinity of a dominant trait variant, particularly so for rare variants. It is important to point out that we focus on local properties of an inherited dominant disease variant, irrespective of other disease variants that might be present on the same or on different chromosomes.

In contrast to previously proposed approaches (Klein et al., 1998), as shown below, we work with average heterozygosities over multiple adjacent markers. Our approach identifies peaks of high variant heterozygosity and surrounding regions of increased variant heterozygosity that are likely to contain pathogenic dominant variants. Unlike linkage analysis, it can be applied to single individuals and to families with a handful of individuals for which linkage analysis would not be very informative, since small linkage blips would be observed throughout the genome. Methods for disease gene mapping have been proposed to combine different approaches (Koboldt et al., 2014). Our method is purely genetic and is extremely useful in conjunction with filtering since the number of variants which would need to be tested is greatly reduced and this would be particularly true when only one individual has been sequenced and/or whole‐genome sequencing has been performed. We anticipate that HA will preferentially be applied to candidate disease variants and will serve as one of the methods to prioritize such variants as has recently been done by the use of HM for recessive traits, where authors required a candidate variant to be located in or near a homozygous region greater than 2 Mb (Sang et al., 2018).

## 2 MATERIALS AND METHODS

### 2.1 Simple population genetics model

To demonstrate that increased heterozygosity around an inherited dominant trait variant is a general phenomenon and not disease‐specific, we assume the following simple model. Consider a dominant disease variant that is successively passed from parents to children. In the course of its segregation through generations, recombination events (due to crossing‐over) will occur to the left and right of the pathogenic variant. In an individual carrying such a (heterozygous) variant, for the chromosome carrying the variant, consider the genomic region, *R*, between the two flanking recombination events closest to the pathogenic variant. This region was passed unchanged from parents to children, that is, the nucleotides in this region of the disease chromosome stay fixed in different generations while variants on the homologous chromosome (not carrying the disease variant) change randomly due to recombination.

At the disease variant, we distinguish two alleles, where + designates the wild‐type allele and *u* the mutant allele. Consider a variant with alleles *A* and *B* in the *R* region. Population frequencies for the *u* and *A* alleles are *e* and *f*, respectively, and *D* is the disequilibrium parameter between the two sites (Table 1). Assume that in the *R* region, only the *A* allele occurs on the chromosome carrying the mutant disease allele so that the (*u*−*B*) cell (Table 2) has zero frequency, that is, only three haplotypes exist (*D*’ = 1), and the disequilibrium parameter is given by *D* = *e*(1−*f*) = *D*_max_. With this, an individual is heterozygous when a *B* allele occurs in coupling with the + allele. Thus, the probability of being heterozygous *A*/*B* at the variant in region *R* is given by:

$$H\;=P(B|\;+,D\;=D_{\max})\;=\left(1-f\right)\;/\left(1-e\right)\;>1\;-f.$$

**Table 1:** Rankings of *H* (Stage 1) and *H*_max_ (Stage 2) values for each individual analyzed and for family members analyzed jointly

	Stage 1 (*H* values)			Stage 2 (*H*_max_ values)				
	Rank	top%	*N*_var_	Rank	d	Rank2	Rank3	Rank4
S1; A, C	199,591	3.1	6,347,882	268	553	252	228	970
S5; A, C	78,924	1.2	6,347,882	480	533	464	420	138
S9; U, C	287,746	4.5	6,347,882	468	20	452	432	141
S2; U, N	1,889,519	29.8	6,347,882	366	119	354	332	1046
S6; U, N	1,148,993	18.1	6,347,882	994	1,429	978	923	279
S7; A, N	709,184	11.2	6,347,882	587	8	577	557	1176
S1, S5, S7; A	38,654	0.6	6,347,882	298	0.7	282	254	66
S1, S5, S9; C	78,225	1.2	6,347,882	516	3	494	458	1706
S2, S6, S7; N	207,214	3.3	6,347,882	394	115	374	342	102
M1; A, C	87,720	20.7	424,175	66	7,432	66	60	26
M2; A, C	126,896	29.5	430,739	58	7,497	56	48	18
M7; U, C	87,868	18.3	481,206	32	7,618	30	24	10
M9; A, C	77,205	20.1	383,563	26	2,238	26	20	10
M1,2,7,9; C (1)	39,632	3.7	1,082,429	124	67	111	81	33
M1,2,7,9; C (4)	6,010	5.9	102,321	25	2,285	20	16	11
L21; A, C	1,305,196	20.4	6,395,904	138	555	105	78	218
L22; A, C	172,382	2.9	5,996,970	84	556	57	39	18
L21,22; C (1)	318,778	3.0	10,549,605	1,898	0.2	1,194	768	2,896
L21,22; C (2)	133,870	7.3	1,843,269	93	553	73	205	172
*False positives*								
Ctrl; BRCA2	131,040	22.0	594,234	66	66,596	66	56	26
Ctrl; WFS1	242,130	40.6	594,234	*n*	*n*	*n*	*n*	*n*
Ctrl; PHOX2	423,768	71.0	594,234	40	5,904	40	31	16
M9; NCBI1	223,045	58.2	383,563	26	15,889	26	20	10
M9; NCBI3	179,653	46.9	383,563	26	16,062	26	20	10

Family S contains three affected females (S1, S5, and S7), two unaffected noncarriers (S2 and S6), and one unaffected carrier (S9) of the BRCA2 pathogenic variant in this family. In family M, individuals M1, M2, and M9 are affected, and M7 is an unaffected obligate carrier. Symbols: A  =  affected, U  =  unaffected, C  =  carrier, N  =  noncarrier, *N*_var_  =  number of variants (*H* values), *d*  =  absolute difference in kb between estimated and true position of the pathogenic variant, *rank2*  =  rank given that the RIH (region of increased heterozygosity) segment length is at least 25% of the average of all RIH lengths; *rank3*  =  rank given that RIH length is at least 50% of the average RIH length; *rank4*  =  rank given that RIH length is at least equal to the average RIH length; *n* at Stage 2 means that the *H* value for the known pathogenic variant does not occur in any RIH. *False positives*: First item  =  individual (Ctrl  =  unaffected in psoriasis family 12), second item  =  assumed disease variant (NCBI: intronic variants).

**Table 2:** Standard parameter settings for haplotypes at a disease variant and a nearby marker variant

	Marker variant		
Disease variant	*A*	*B*	Sum
*u*	*ef*+*D*	*e*(1−*f*)−*D*	*e*
+	(1−*e*)*f*−*D*	(1−*e*)(1−*f*) + *D*	1−*e*
Sum	*f*	1−*f*	1

Symbols: *e  *=*  *disease allele (mutation) frequency, P(*u*); *f  *=* * marker variant allele frequency, P(*A*); *D * =* * disequilibrium parameter

Thus, such a variant with low minor allele frequency (MAF), *f*, is highly likely to be heterozygous. On the other hand, at an analogous variant far away from the disease variant (outside the *R* region, or on a different chromosome), the probability of being heterozygous is given by 2 *f*(1−*f*), which is small for small *f*. As *e* is assumed to be a small number, heterozygosity in region *R* is larger than in a random region. The ratio of heterozygosity in the *R* region over heterozygosity in a random region is given by *r* = 1/[2 *f* (1−*e*)] > 1/(2 *f*), which can be rather high, particularly for small variant allele frequencies, *f*, which are of most interest. Clearly, this model is very simple but it unequivocally demonstrates increased marker heterozygosity around an inherited dominant pathogenic variant.

As recombination events occur relatively randomly along a chromosome, the length of the *R* region will depend on the number of recombinations experienced by a chromosome. Therefore, a pathogenic variant transmitted from parents to children over many generations is expected to be located in a short R region. Conversely, a newly mutated pathogenic variant will not show increased heterozygosity of variants surrounding it. However, there are various factors other than the age of a pathogenic variant that can influence the length of the *R* region, for example, local LD.

### 2.2 Average heterozygosity

The main determinant of a variant's heterozygosity is its allele frequency. To mitigate the effects of varying allele frequencies and missing genotypes, at each variant we compute a moving average for heterozygosity and then follow these averages across each chromosome. As outlined below, we proceed in two stages.

At Stage 1, at each variant throughout the genome, we compute an average heterozygosity, *H*. For example, for a 101‐point average at a given variant, we consider it and the 50 variants on either side and determine the proportion *H* of heterozygous genotypes in one or more individuals. Thus, the first and last 50 variants on each chromosome will not have an *H* value associated with it. Specifically, a (2 *m*+1)‐point average *H* is obtained by counting the number *g* of homozygous genotypes and the number *h* of heterozygous genotypes at the given variant and the *m* variants on either side of it. Analogous counts would be obtained when *n* family members are analyzed together. Heterozygosity is then obtained as *H* = *h*/(*g*+*h*). Without missing genotypes and *n* individuals analyzed jointly, the total number of genotypes would be (2 *m*+1)*n* = *g* + *h*. Thus, on each chromosome, the first and last *m* variants will not obtain an *H* value.

At Stage 2, to focus on high *H* values at a good number of consecutive variants, we form regions of increased heterozygosity, RIH, such that *H* values keep increasing up to a maximum, *H*_max_, and then decrease again. For a given chromosome, consider an ordered set of *H*_i_ values, *i* = 1…*k*. We now form successive differences, *C*_i_ = *H*_i_ – *H*_i‐1_, for *i* = 2…*k*. Negative values of *C* signal an increase and positive values a decrease in *H*. Thus, we search for the first *C*_i_ < 0 and keep going until *C* turns positive (at *H*_max_); we keep going as long as *C* values stay positive until we reach *C*_j_ < 0. In this manner, we find an RIH segment ranging from variants with *C*_i_ through *C*_j_. Values of *C* = 0 are skipped in this process.

Calculations described above for stages 1 and 2 have been implemented in a computer program, *PH*, which is available for Windows and Linux computers (http://lab.rockefeller.edu/ott/programs).

### 2.3 Significance testing

Previous attempts at disequilibrium mapping for dominant traits (Klein et al., 1998) may have been hampered by low informativeness of single marker variants. As mentioned above, we use average marker heterozygosity for at least 101 adjacent markers. While the use of single markers allows derivation of elegant test statistics (Klein et al., 1998), this does not seem possible for our approach, so we resort to computer‐based estimation of empirical significance levels. Assuming as the null hypothesis that the disease variant can be at any of the marker positions in the dataset, we randomly place the disease variant at one of the *n* marker positions, where *n* is the total number of markers used in the analysis; for example, *n* = 6,347,882 for individuals in the S family. With a given null position, we carry out analysis as done for the observed data and record the value *H* at the null disease variant and the *H*_max_ value in the segment containing the null disease variant. This completes one replicate in our computer‐based procedure. Based on the *H* values obtained in *N* replicates and the observed value, *H*_obs_, we count the number *k* of values at least as large as *H*_obs_ and obtain the empirical significance level associated with *H*_obs_ as *p* = *k*/(*N*+1). Analogous calculations are done for *H*_max_.

## 3 RESULTS

### 3.1 Family data

**Figure 1:**
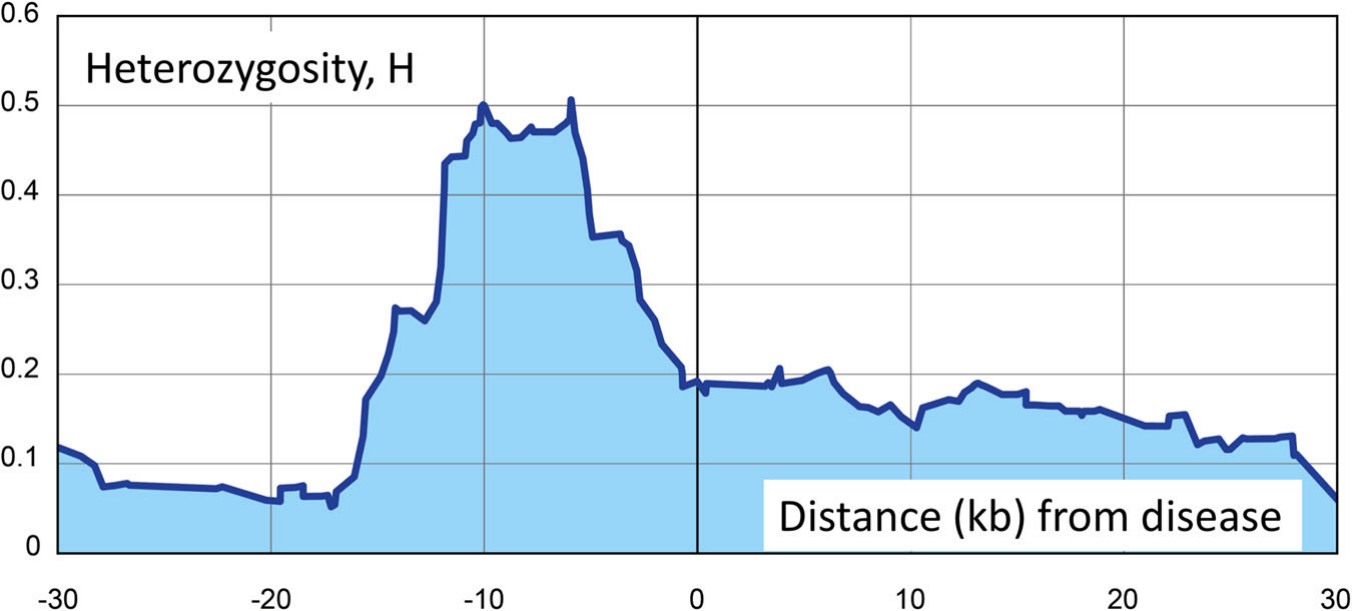
Stage 1 heterozygosity rate, *H* (*y*‐axis), plotted against marker positions (*x*‐axis) surrounding a pathogenic *BRCA2* mutation in three females affected with breast cancer in family S. The *x*‐axis scale is the distance in kb from the pathogenic *BRCA2* mutation

**Figure 2:**
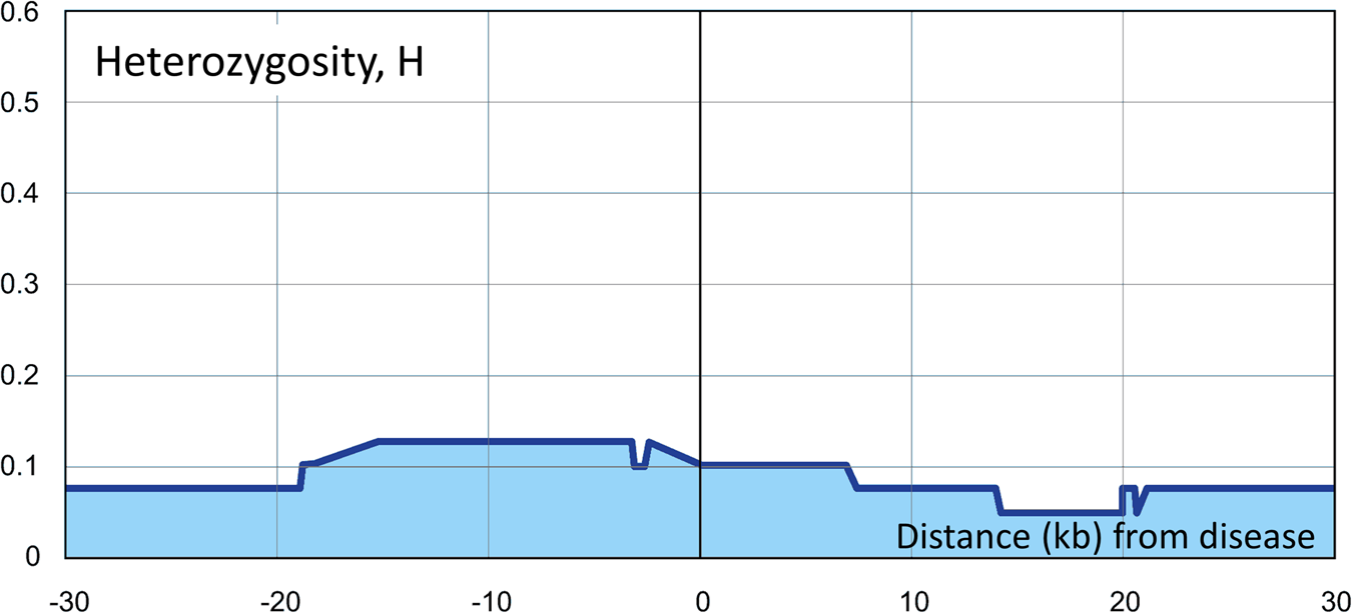
Stage 1 heterozygosity rate, *H* (*y*‐axis), plotted against marker positions (*x*‐axis) surrounding a pathogenic *BRCA2* mutation in a control individual, that is, the first individual in our unpublished collection of family members affected with psoriasis. The *x*‐axis scale is the distance in kb from the pathogenic *BRCA2* mutation

We applied our approach to three dominant traits with known pathogenic variants, breast cancer in family S (Ataei‐Kachouei et al., 2015; BRCA2 gene), neuroblastoma in family M (McConville, Reid, Baskcomb, Douglas, & Rahman, 2006; Mosse et al., 2004; PHOX2B gene), and low‐frequency nonsyndromic hearing impairment in family L (Bespalova et al., 2001; WFS1 gene). Family graphs are provided as Figures S1, S2, and S3, respectively, and technical details on sequencing are provided in Supporting Information. For each trait, one family with multiple individuals was investigated. As an example, for all affected members of family S, Figure 1 shows *H* values (Stage 1) on the ordinate (*y*‐axis) at variants within +/− 30 KB of the BRCA2 pathogenic variant, rs276174825, on chromosome 13. Clearly, variant heterozygosity is increased in the vicinity of the pathogenic variants. Analogous figures occur for the other families (not shown here) and have somewhat varying shapes in different families, depending on variant densities and sequencing properties. For comparison, for the first individual in our unpublished collection of family members affected with psoriasis, Figure 2 shows the rather unremarkable curve of *H* values, obtained in analogy to those in Figure 1. As a pseudo‐disease variant, we chose the variant in that dataset closest to the pathogenic variant in the BRCA2 gene on chromosome 13. The resulting Stage 1 rank of this pseudo‐disease variant was 277,393 (top 37%) among all 742,520 *H* values.

Based on these variant‐specific *H* values, RIH segments of increasing and then decreasing *H* values were created at Stage 2, with each segment containing a local *H*_max_ value. Thus, both stages furnish heterozygosity values, *H* at Stage 1 and *H*_max_ at Stage 2, where the latter may be used for prioritizing variants as candidates for pathogenic disease variants. As will be shown below, while *H*_max_ values serve to identify the approximate location of a disease variant, the top % rankings of the associated *H* values can discriminate between true and false peaks. Table 1 shows rankings for each analyzed individual and for families analyzed as a whole. For Stage 1, the *H* value of the pathogenic variant was ranked among *H* values at all variants, where the largest *H* value is ranked 1. For Stage 2, the *H*_max_ value in the RIH segment containing the pathogenic variant was ranked among all *H*_max_ values. It is immediately clear that *H*_max_ values are much better predictors than *H* values. It is also obvious that analyzing multiple individuals in a family is better than analyzing one individual at a time.

Rankings of *H*_max_ may be improved by disregarding *H*_max_ values in RIH segments that are unusually short. One might expect that an RIH segment containing a pathogenic disease variant should be longer than the average of all RIH segments. However, our analysis has shown (not detailed here) that this is not always the case. Thus, we tested four different ways of ranking *H*_max_ values: All *H*_max_ values (“rank” in Table 1, Stage 2); *H*_max_ values in RIH segments longer than *w*/4, where *w* is the average length of all RIH segments (“rank2”); *H*_max_ values in RIH segments longer than *w*/2 (“rank3”); and *H*_max_ values in segments longer than *w* (“rank4”). Ranking all *H*_max_ values is most conservative while ranking based on “rank4” values is most risky in the sense that RIH segments may be disregarded that contain the pathogenic variant. If this happens then ranking can be worse than when all *H*_max_ values are used as may be seen, for example, for individual S1 in Table 1. In our data, applying the “rank2” criterion has always been better than ranking all *H*_max_ values. Thus, we recommend as a rather safe approach to disregard RIH segments smaller than *w*/4 and rank the *H*_max_ values in the remaining RIH segments. The pathogenic variant was generally found to be close to one of the top few hundred best‐ranked *H*_max_ values. For families S and L, the highest ranked variants were within a distance *d* of a few kb base pairs although, for unknown reasons, this distance was considerably larger (*d* = 237 kb) for the affected and obligate carriers in family M (M1, M2, M7, M9).

### 3.2 Statistical significance

Based on *N* = 999 random replicates, genome‐wide empirical significance levels, *p*, were obtained for observed *H* and *H*_max_ values in each individual in families L, M, and S, with the smallest possible value being *p* = 1/1000 = 0.001. None of the *H*_max_ values furnished significant results (*p* < 0.05) while several *H* values were highly significant: In family S, all individuals carrying the pathogenic BRCA2 mutation (“carrier” in Table S1) show *p* < 0.05 for their *H* values and all noncarriers show *p* > 0.10. Evidently, *H* values have the potential to discriminate between carriers and noncarriers; this issue is evaluated more formally in the next section. One individual, S7, is an affected noncarrier, yet shows *p* = 0.019. Thus, it appears likely that this individual's affection status is caused by a mutant variant close to the known BRCA2 pathogenic variant investigated here but this was not further pursued. Results for families L and M are largely nonsignificant (Table S1).

### 3.3 Discriminating between true and false positive results

As shown above and demonstrated in Table 1, *H*_max_ values are highly suitable for prioritizing marker variants as candidates for inherited dominant pathogenic variants. For example, out of over 6 million variants in family S, *H*_max_ values in the segments containing the known disease variants rank in the top few hundred, and in family M, out of over 400,000 variants, less than 100 variants show an *H*_max_ rank higher than that of the known pathogenic variant. However, as *H*_max_ values have been maximized (they represent the largest of all *H* values in an RIH segment), they also tend to be relatively high in noncarrier individuals even though average ranks in carriers is lower (better) than in noncarriers. For example, the three carrier and the three noncarrier individuals in family S (Table 1) show respective average *H*_max_ ranks of 405.3 and 649.0. Nonetheless, *H*_max_ values are not good for discriminating between true and false positive results (see below). On the other hand, we show here that *H* values, not being maximized, are well suited for discriminating true from false results. To demonstrate this feature, we relied on our relatively small sample of nine known carriers and three known noncarriers (Table 1). To increase the latter number, we used our psoriatic control individual three times, each time pretending that the known pathogenic variants of the BRCA2, WFS1, and PHOX2 genes are disease variants for this individual. In addition, individual M9 was used twice: (a) with a variant in the UTR (position 55,396,207 on chromosome 4; here called “NCBI1”) of the TMEM165 gene, and (b) with an intronic variant (position 55,569,682 on chromosome 4; “NCBI3”) in the PDCL2 gene (all base pair positions in this report refer to the GRCh37.p13 assembly).

**Figure 3:**
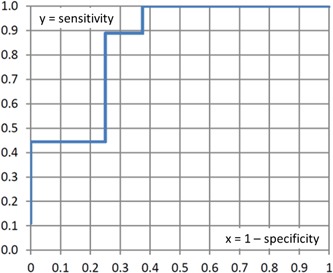
Receiver operating characteristic curve based on results of Table 3. The graph shows *y* = sensitivity plotted against *x* = 1 − specificity. The area under the curve is AUC =0.85

Now we had nine carriers and eight noncarriers and applied our PH program to each of these 17 individuals. Table 3 shows top % results for *H* values. For each of the 17 lines in Table 3, we considered a top % value just below the one on that line as a threshold to predict individuals to be carriers when they have a top % value above this threshold. For example, for line 6, a suitable top % threshold would be 18.2. For each of these 17 thresholds, we estimated sensitivity (proportion of carriers predicted to be carriers) and specificity (proportion of noncarriers predicted to be noncarriers). For example, there are six individuals with a top % value below the threshold of 18.2, so these would be predicted to be carriers while the 11 individuals exceeding the threshold would be predicted to be noncarriers. In reality, among all nine carriers, based on this threshold, four are predicted to be carriers and five are predicted to be noncarriers, so sensitivity =4/9 = 0.444. As is customary, we graphed *y* = sensitivity against *x* = (1 − specificity). The resulting receiver operating characteristic (ROC) curve is shown as Figure 3. The area under the curve, AUC, is generally considered a measure for discriminating power, where AUC =0.5 refers to no discrimination. Despite our small sample size, we obtained a respectable AUC =0.85. Thus, based on this admittedly very small sample, a reasonable rule would be to declare a candidate variant a true positive when analysis for a given individual or family results in an *H* value with associated top % smaller than 21%, which is estimated to have sensitivity of 0.889 and specificity of 0.750 (the corresponding total probability of correct prediction is estimated to be 0.824, the highest value for these data). It is planned to collect many more known carriers and noncarriers for various pathogenic disease variants so as to make prediction as accurate as possible.

**Table 3:** Nine carriers (C) and eight noncarriers (N) and top % results for *H* values of disease variants and pseudo‐disease variants, respectively

ID	*H* top %	Status	Sensitivity	Specificity
S5	1.2	C	0.111	1
L22	2.9	C	0.222	1
S1	3.1	C	0.333	1
S9	4.5	C	0.444	1
S7	11.2	N	0.444	0.875
S6	18.1	N	0.444	0.750
M7	18.3	C	0.556	0.750
M9	20.1	C	0.667	0.750
L21	20.4	C	0.778	0.750
M1	20.7	C	0.889	0.750
Ctrl; BRCA2 disease variant	22.0	N	0.889	0.625
M2	29.5	C	1	0.625
S2	29.8	N	1	0.500
Ctrl; WFS1 disease variant	40.6	N	1	0.375
M9; NCBI3	46.9	N	1	0.250
M9; NCBI1	58.2	N	1	0.125
Ctrl; PHOX2 disease variant	71.0	N	1	0

We constructed an ROC curve also for *H*_max_ values associated with the *H* values used in the previous paragraph (details not shown) and obtained a value of AUC = 0.60. Clearly, *H* values are much better discriminators than *H*_max_ values, which is in line with our findings in the paragraph on statistical significance in Section 3.

### 3.4 Population data

We also investigated a collection of control individuals, the ALSPAC dataset (Boyd et al., 2013; Fraser et al., 2013) of 1,927 population individuals who had been whole‐genome sequenced (ascertainment and study numbers are provided in Supporting Information). The ALSPAC study website contains details of all the data that are available through a fully searchable data dictionary and variable search tool (http://www.bristol.ac.uk/alspac/researchers/our‐data/). Rather than identifying individuals affected with a genetic condition, we were looking for relatively common dominant pathogenic variants to identify individuals in this dataset carrying such variants. To keep the total number of variants to a manageable level, we focused on the *n* = 3,758,237 variants on chromosome 2. In the OMIM (https://www.ncbi.nlm.nih.gov/omim) database, we looked up dominant pathogenic variants on chromosome 2 and found 357 of them with their chromosomal positions. However, only 10 positions were present in the ALSPAC dataset.

Using our PH program, for all individuals combined (phenotypes = “unaffected”), we computed *H* and *H*_max_ values for each of these 10 dominant pathogenic variants. Resulting largest *H* top% values ranged from 2.1 (variant rs3214759 in the CRYGB gene) to 96.8% (rs2020912 in the MSH6 gene). According to the OMIM database, rs3214759 is involved in various forms of dominantly inherited cataract (OMIM number 615188). As Figure S4 shows, the graph of *H* values around the pathogenic variant rs3214759 shows a peak very similar to the one seen for breast cancer families (Figure 1), which confirms the inherited nature of this pathogenic variant.

As a negative control, we wanted to work with a nonpathogenic variant. The CRYGB gene is only 3.58‐kb long, so we were looking for variants 10 MB upstream (far away) of the CRYGB gene and identified rs1978890, a common intron variant in the PLCL1 gene without known functional consequences. Figure S5 demonstrates absence of a peak of *H* values around this seemingly neutral variant.

These observations in the ALSPAC dataset demonstrate that our approach is not only useful in family data but can also be successfully applied to population data. For the pathogenic variant rs3214759 for cataracts, at Stage 2, 1,756 RIH segments were formed, each containing a local *H*_max_ value. One segment contains rs3214759, whose *H*_max_ value ranks 118 and is located (estimated position of the disease variant) 45 kb away from rs3214759 while the peak *H* value close to rs3214759 is only 277 bp from it.

## 4 DISCUSSION

There are several similarities and differences between HA developed here for dominant traits and HM previously published (Lander & Botstein, 1987) for recessive traits. One important similarity is that both can be used on single individuals; when multiple (affected) individuals are analyzed, their relationships, if any, need not be known. On the other hand, HM relies on linkage and furnishes long ROHs while the basis for HA is LD between marker and disease variants, and LD is known to dissipate quickly with increasing distance between two variants, which has previously been documented (Genomes Project et al., 2015; Hartl & Clark, 1989). Consequently, our HA approach can be expected to furnish rather precise results, and this is likely to hold also in the presence of multiple disease‐causing variants throughout the genome. With three or more affected individuals, if there is a single pathogenic variant in the exome, one may have only a few variants in the selected region which can be further reduced by removing those variants which are not rare, for example, MAF <0.005 in all populations in *gnomAD* (Lek et al., 2016; Zhang et al., 2017) and by biomathematics evaluation. In this situation, when exomes are analyzed, our approach may be only somewhat superior to current filtering approaches, since for three affected individuals only a limited number of variants will be shared. However, HA becomes very important for analyzing single individuals and variants which lie outside of the coding region as it will greatly reduce the number of variants since the space where the pathogenic variant lies is reduced from the genome to a small genomic region. An additional benefit of this method is that it can aid in the detection of locus heterogeneity within a pedigree. For multiple affected family members, it can be observed whether or not they share a region of heterozygosity. This information can then be used to inform the filtering process.

In HM, low MAF of a variant is a major confounder as it tends to render variants homozygous just because of allele frequency. On the other hand, in HA, variants with a generally low MAF are highly likely to be heterozygous when they are close to an inherited dominant pathogenic variant, which represents a major difference between HM and HA.

One might have expected that runs of heterozygous variants could be found by tricking one of the HM procedures into focusing on heterozygous rather than homozygous variants. We tried such an approach with the HM algorithm implemented in *plink* (Purcell et al., 2007) and for individual S1 changed all homozygous variants to being heterozygous and vice versa. However, *plink* reported that no “ROH” was found, and this did not change even when we tried different parameter settings to make it easy for *plink* to detect runs of heterozygosity, so we did not pursue this avenue further.

In addition to *H* and *H*_max_ values, we considered several other statistics and their performance for prioritizing variants, for example, the length *L* of each RIH (containing an *H*_max_ value each), and *H*_max_ weighted by *L*. For our data with known pathogenic variants, *H*_max_ consistently showed the highest ranks, particularly when *H*_max_ values in short RIH segments are disregarded.

As shown in Section 2, results depend somewhat on the choice of *N*_avg_, that is, what value is used at Stage 1 for *N*_avg_‐point moving averages of *H*. However, this sensitivity is not unique to our approach. For example, results of HM as implemented in *plink* (Purcell et al., 2007) depend on at least four different parameters.

The approach presented here might be improved by working with map positions instead of base‐pair positions (Li et al., 2015). A given distance in bp has a different meaning in areas of low and high LD. Working on a map with constant LD (Tapper et al., 2005) would presumably be particularly beneficial at Stage 2 as it would make lengths of RIHs more comparable in different genomic areas, but this has not been investigated in this context. Another potential improvement might be to assign weights to variants based on their allele frequencies although their effects might be minimized by our use of *n*‐point averages for *H* values.

## CONFLICT OF INTERESTS

The authors declare that there are no conflicts of interests.

## DATA ACCESSIBILITY

Software developed for our HA analysis is freely available from http://lab.rockefeller.edu/ott/programs. The download package contains the programs *prophet* and *negpos*, combined into a shell program, *PH*, and sample data.

All vcf files (other than those from the ALSPAC collection) used in our manuscript have been deposited with the European Genome‐phenome Archive (EGA), Wellcome Trust Genome Campus, Hinxton, Cambridge CB10 1 SD, UK, with study accession number EGAS00001002837.

## SUPPORTING INFORMATION

Three Figures, S1, S2, and S3 show graphs of the three families. Figures S4 and S5 show graphs of *H* values surrounding known dominant pathogenic variants rs3214759 in gene CRYGB (cataract) and rs74874677 in gene DGUOK, respectively. Also, a section on genotype data provides detailed information on sequencing methods, and ascertainment and study numbers for the ALSPAC dataset is given in a separate section. Table S1 displays results of genome‐wide statistical significance tests.

## Supporting information

Supporting information
