# Supplementary material for: Heterozygosity mapping for human dominant trait variants
Source: Hum Mutat. 2019 Apr 24;40(7):996–1004. doi: 10.1002/humu.23765 (PMC6617796; doi:10.1002/humu.23765)
Supplement: Supplementary file 1 — Supporting information [file HUMU-40-996-s001.pdf]

Heterozygosity mapping for human dominant trait variants

Atsuko Imai-Okazaki, Yi Li, Sukanya Horpaopan, Yasser Riazalhosseini, Masoud Garshasbi, Yael P. Mosse, Di Zhang, Isabelle Schrauwen, Aarushi Sharma, Cathy S. J. Fann, Suzanne M. Leal, Mark Lathrop, and Jurg Ott

Supporting Information

Family graphs

The three supp. Figures S1, S2, and S3 show graphs of families S (Ataei-Kachouei et al., 2015), M (McConville, Reid, Baskcomb, Douglas, & Rahman, 2006; Mosse et al., 2004), and L (Bespalova et al., 2001), respectively.

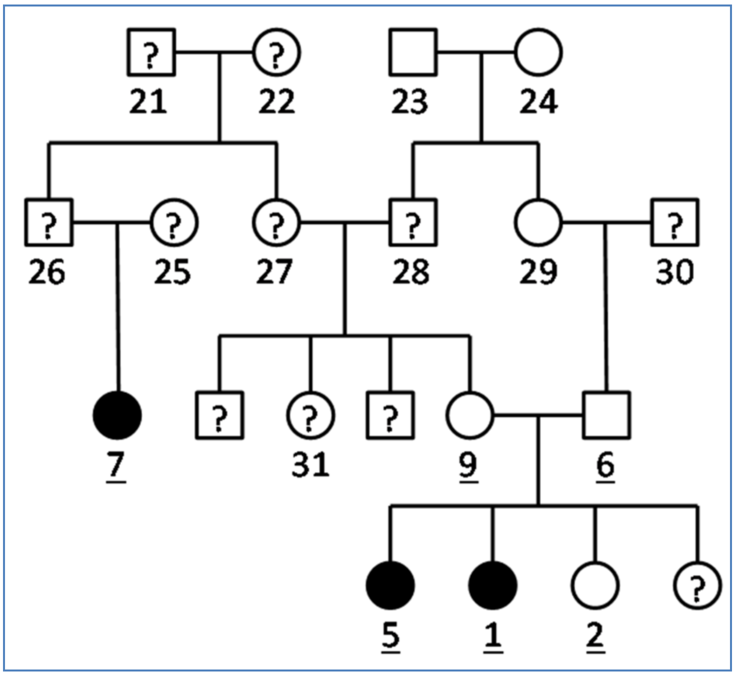

Supp. Figure S1: Family S (see Table 1 in main paper)

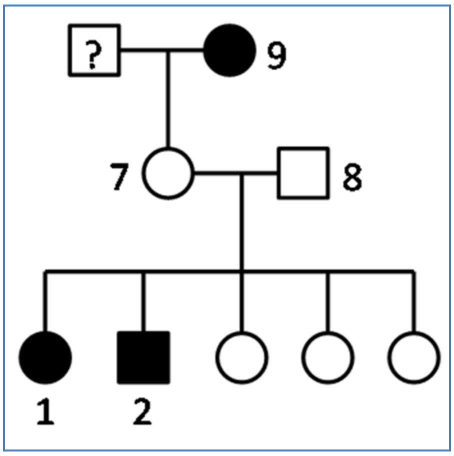

Supp. Figure S2: Family M. Individuals 1, 2, 7 (obligate carrier), and 9 had been sequenced and were used in our analysis.

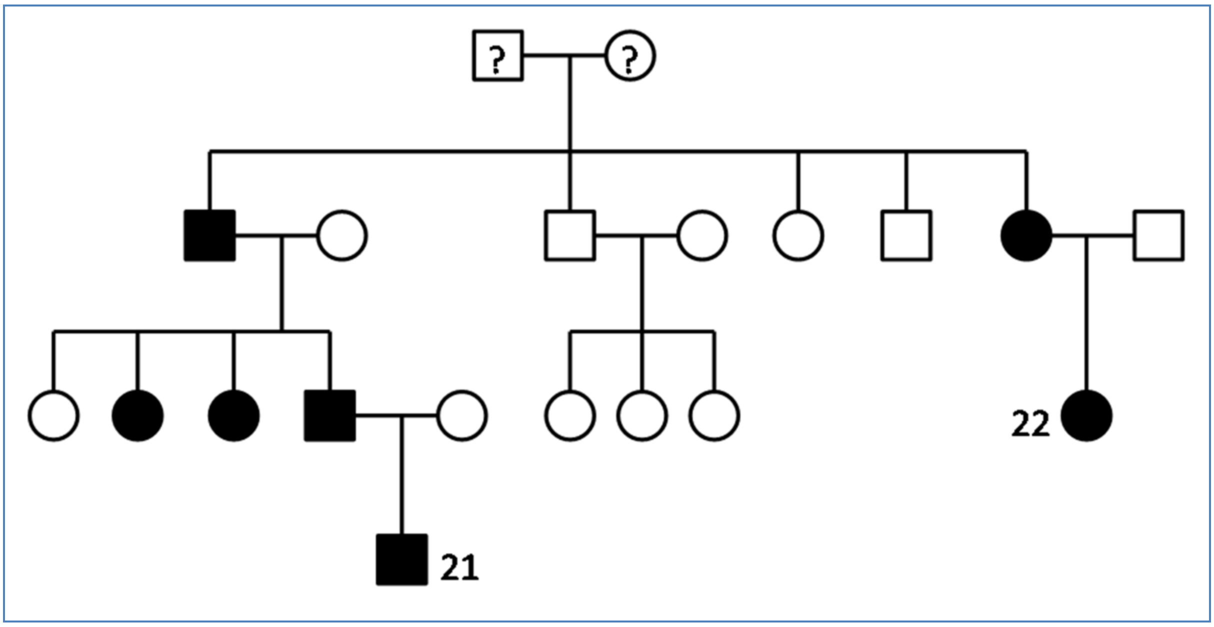

Supp. Figure S3: Family L. Individuals labeled "21" and "22" had been sequenced and are the only two individuals used in our analysis.

Family genotype data

The data for families S (Ataei-Kachouei et al., 2015) and L (Bespalova et al., 2001) have previously been published but sequencing for family L was only recently done for educational purposes. Family M has not previously been published but is one of a number of families with the same pathogenic variant (Mosse et al., 2004) and has recently been sequenced for instructional purposes. Details on sequencing may be found in the references in this paragraph, and in reference (Frampton et al., 2013) for family M. Data were furnished to us in the form of *vcf* files and were converted to *plink* transposed format with the following parameters:

```
--const-fid
--allow-extra-chr
--chr 1-22
--biallelic-only
--maf 0.00000001
--output-missing-phenotype 0
--vcf-min-qual 10
--recode 12 transpose
```

The data contained a small fraction of indels (the pathogenic variant in family S is an indel). All individuals for family S were contained in the same *vcf* file with 6,347,882 variants; the pathogenic variant reported here is the only disease variant in the BRCA2 gene in this family. Files for family M were available individually and resulted in approximately 430,000 variants for each of the four individuals. For family L, the two individuals #21 and #22 furnished 45,506 and 44,646 variants, respectively. Thus, these variants were not used and the two vcf files were re-read without the "minimum quality" qualifier, which resulted in 6,395,904 and 5,996,970 variants, respectively. To analyze members of families M and L combined for each family, individual *plink* genotype files were merged so that only those variants were retained with a known genotype for each individual, which resulted in 115,307 and 1,843,269 variants for families M (three affecteds) and L (two affecteds), respectively. No other filtering was applied.

ALSPAC study numbers

Pregnant women resident in Avon, UK with expected dates of delivery 1st April 1991 to 31st December 1992 were invited to take part in the study. The initial number of pregnancies enrolled is 14,541 (for these at least one questionnaire has been returned or a "Children in Focus" clinic had been attended by 19/07/99). Of these initial pregnancies, there was a total of 14,676 foetuses, resulting in 14,062 live births and 13,988 children who were alive at 1 year of age.

When the oldest children were approximately 7 years of age, an attempt was made to bolster the initial sample with eligible cases who had failed to join the study originally. As a result, when considering variables collected from the age of seven onwards (and potentially abstracted from obstetric notes) there are data available for more than the 14,541 pregnancies mentioned above.

The number of new pregnancies not in the initial sample (known as Phase I enrolment) that are currently represented on the built files and reflecting enrolment status at the age of 18 is 706 (452 and 254 recruited during Phases II and III respectively), resulting in an additional 713 children being enrolled. The total sample size for analyses using any data collected after the age of seven is therefore 15,247 pregnancies, resulting in 15,458 foetuses. Of this total sample of 15,458 foetuses, 14,775 were live births and 14,701 were alive at 1 year of age.

A 10% sample of the ALSPAC cohort, known as the Children in Focus (CiF) group, attended clinics at the University of Bristol at various time intervals between 4 to 61 months of age. The CiF group were chosen at random from the last 6 months of ALSPAC births (1432 families attended at least one clinic). Excluded were those mothers who had moved out of the area or were lost to follow-up, and those partaking in another study of infant development in Avon.

Population data

Supp. Figures S4 and S5 below refer to the 1,927 individuals in the ALSPAC data analyzed by our approach. We focused on pathogenic dominant variants on chromosome 2 (see main paper). Figures S4 and S5 show *H* values in the vicinity of the two pathogenic variants with the best (lowest ranked) *H* values.

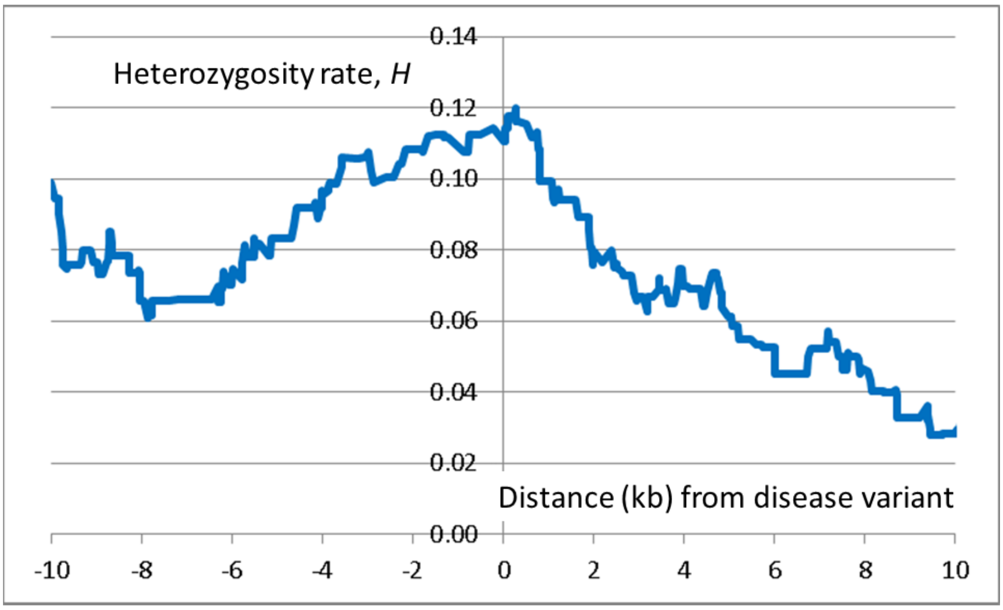

Supp. Figure S4. Curve of *H* values for the pathogenic variant rs3214759 in the CRYGB gene (cataracts). The figure clearly shows the typical peak close to the pathogenic variant in inherited dominant disease variants.

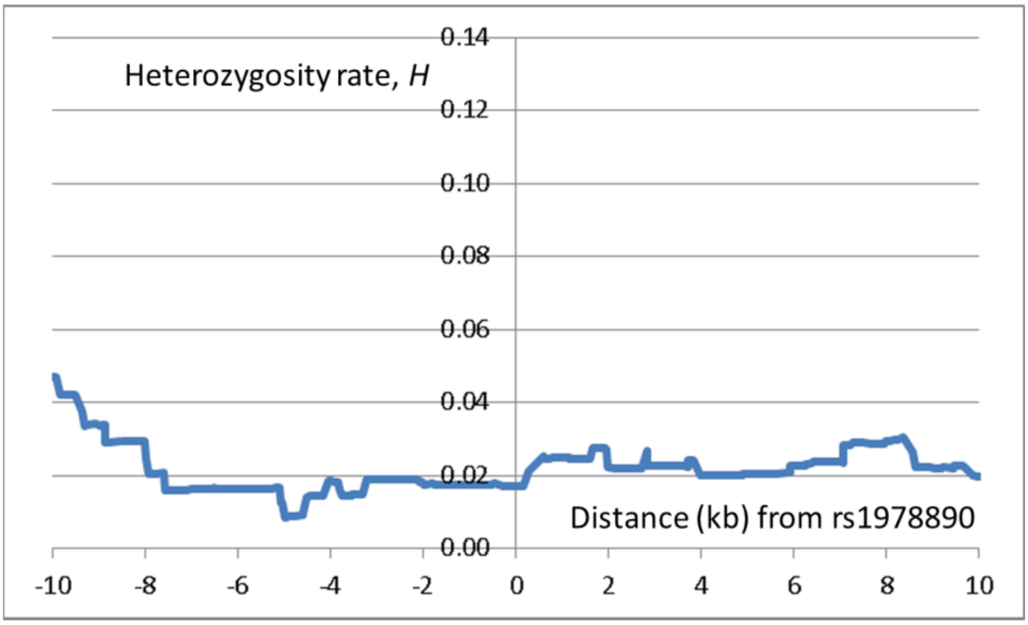

Supp. Figure S5. Curve of *H* values for the common intron variant rs1978890 in the PLCL1 gene. The figure demonstrates absence of a peak close to the variant.

Statistical significance

	H (stage 1)		H_{max} (stage 2)	
Individual	*obs*	*p*	*obs*	*p*
**Family S**				
S1 carrier, affected	0.242	0.037	0.379	0.147
S5 carrier, affected	0.262	0.018	0.310	0.270
S9 carrier, unaffected	0.214	0.046	0.333	0.243
S2 noncarrier, unaffected	0.119	0.310	0.386	0.186
S6 noncarrier, unaffected	0.120	0.183	0.256	0.511
S7 noncarrier, affected	0.337	0.019	0.363	0.310
**Family M**				
M1, carrier, affected	0.439	0.194	0.570	0.564
M2, carrier, affected	0.403	0.273	0.644	0.424
M7, obligate carrier, unaffected	0.450	0.183	0.704	0.225
M9, carrier, affected	0.456	0.199	0.710	0.208
**Family L**				
L21, carrier, affected	0.010	0.314	0.069	0.471
L22, carrier, affected	0.030	0.044	0.079	0.268

Supp. Table S1. Observed ("obs") values of *H* and H_{max} and associated empirical significance levels, *p*, based on 1,000 random replicates.

References

Ataei-Kachouei, M., Nadaf, J., Akbari, M. T., Atri, M., Majewski, J., Riazalhosseini, Y., & Garshasbi, M. (2015). Double heterozygosity of BRCA2 and STK11 in familial breast cancer detected by exome sequencing. *Iran J Public Health, 44*(10), 1348-1352. Retrieved from https://www.ncbi.nlm.nih.gov/pubmed/26576347

Bespalova, I. N., Van Camp, G., Bom, S. J., Brown, D. J., Cryns, K., DeWan, A. T., . . . Lesperance, M. M. (2001). Mutations in the Wolfram syndrome 1 gene (WFS1) are a common cause of low frequency sensorineural hearing loss. *Hum Mol Genet, 10*(22), 2501-2508. Retrieved from https://www.ncbi.nlm.nih.gov/pubmed/11709537

Frampton, G. M., Fichtenholtz, A., Otto, G. A., Wang, K., Downing, S. R., He, J., . . . Yelensky, R. (2013). Development and validation of a clinical cancer genomic profiling test based on massively parallel DNA sequencing. *Nat Biotechnol, 31*(11), 1023-1031. doi:10.1038/nbt.2696

McConville, C., Reid, S., Baskcomb, L., Douglas, J., & Rahman, N. (2006). PHOX2B analysis in non-syndromic neuroblastoma cases shows novel mutations and genotype-phenotype associations. *Am J Med Genet A, 140*(12), 1297-1301. doi:10.1002/ajmg.a.31278

Mosse, Y. P., Laudenslager, M., Khazi, D., Carlisle, A. J., Winter, C. L., Rappaport, E., & Maris, J. M. (2004). Germline PHOX2B mutation in hereditary neuroblastoma. *Am J Hum Genet, 75*(4), 727-730. doi:10.1086/424530
